# Identification of different malaria patterns due to *Plasmodium falciparum* and *Plasmodium vivax* in Ethiopian children: a prospective cohort study

**DOI:** 10.1186/s12936-016-1253-2

**Published:** 2016-04-14

**Authors:** Dinberu Seyoum, Yehenew Getachew Kifle, Virginie Rondeau, Delenasaw Yewhalaw, Luc Duchateau, Angel Rosas-Aguirre, Niko Speybroeck

**Affiliations:** Institute of Health and Society (IRSS), Université catholique de Louvain, Brussels, Belgium; Department of Statistics, Natural Science College, Jimma University, Jimma, Ethiopia; Department of Statistics and Operations Research, University of Limpopo, Polokwane, 0727 South Africa; INSERM EMI 0338 (Biostatistics), Université Victor Segalen Bordeaux 2, 146 rue Leo Saignat, 33076 Bordeaux Cedex, France; Department of Laboratory Technology Science and Pathology, College of Public Health and Medical Science, Jimma University, Jimma, Ethiopia; Department of Comparative Physiology and Biometrics, Faculty of Veterinary Medicine, Ghent University, Ghent, Belgium; Institute of Tropical Medicine “Alexander von Humboldt”, Universidad Peruana Cayetano Heredia, Lima, Peru

**Keywords:** Frailty model, Recurrent malaria, *Plasmodium vivax*, *Plasmodium falciparum*, Ethiopia

## Abstract

**Background:**

The identification of epidemiological pattern of infection with *Plasmodium falciparum* and *Plasmodium vivax* in malaria-endemic area, where multiple episodes are common, is important for intervention programmes.

**Methods:**

A longitudinal cohort study based on weekly house-to-house visits was conducted between July 2008 and June 2010 in 2040 children less than 10 years of age, living nearby the Gilgel-Gibe hydroelectric power dam reservoir in order to determine factors associated with increased *P. vivax* and *P. falciparum* incidence. Two types of multivariate frailty models were applied (using time-to-first malaria episode data and time-to-recurrent malaria episode data), allowing the estimation of adjusted hazard ratios (AHR) of potential risk factors (gender, age, proximity to the dam reservoir, and season) for species-specific malaria incidence.

**Results:**

Of 2040 children in 96 weeks of follow up, 864 children experienced at least one malaria episode: 685 due to *P. falciparum* in 548 children, and 385 due to *P. vivax* in 316 children. *Plasmodiumvivax* and *P. falciparum* malaria incidence rates were 8.2 (95 % CI: 7.3–9.1) and 14.6 (95 % CI: 13.4–15.6) per 1000 children per month, respectively. According to the time-to-recurrent event models, children aged ≥7 years had a lower risk of presenting *P. vivax* episodes (AHR = 0.6; 95 % CI: 0.4–0.9), but a higher risk of *P. falciparum* episodes, when compared with children under ≤3 years (AHR = 1.2; 95 % CI: 1.1–1.6). In addition, *P. vivax* (AHR = 2.7; 95 % CI: 2.2–3.5) and *P. falciparum* (AHR = 16.9; 95 % CI: 14.3–20.2) episodes were respectively 2.7 and 16.9 times more frequent in the dry season than in the long rainy season.

**Conclusions:**

The analysis of all malaria episodes (first and recurrent episodes) in the malaria cohort suggests different species-specific patterns of malaria disease in children, with mild seasonality in the incidence of *P. vivax* episodes mostly observed in younger age groups, and with marked seasonality in the incidence of *P. falciparum* episodes mainly seen in older children.

**Electronic supplementary material:**

The online version of this article (doi:10.1186/s12936-016-1253-2) contains supplementary material, which is available to authorized users.

## Background

Despite the good progress over the past decade, malaria remains the most important human vector-borne disease worldwide [1]. According to latest estimates, globally 214 million cases and 438,000 deaths occurred in 2015, mainly among children under 5 years of age living in Africa [1]. Malaria is a major public health problem in Ethiopia, threatening about 70 % of the total population and contributing 4 % to all cases in Africa [2]. Unlike in most endemic African countries [3], both *P. falciparum* and *P. vivax* substantially contribute to malaria morbidity in Ethiopia, accounting respectively for 60 and 40 % of the total reported cases [4]. Species-specific malaria transmission is influenced by climate, topography, human settlement and population movement patterns; and can vary widely across country regions [5, 6].

Unlike *P. falciparum*, *P. vivax* can cause relapses due to its ability to produce latent parasite stages (hypnozoites) [7, 8]. This particular biological characteristic makes it challenging to understand *P. vivax* malaria transmission, given that *P. vivax* recurrent episodes can be caused either by hypnozoite-triggered relapses, resurgence of erythrocytic parasites (i.e. recrudescence) due to a failure in the treatment, or reinfection of individuals with a new parasite strains following primary infections [9].

A cross-sectional community-based study conducted in south-western Ethiopia in 2005 reported increased *P. falciparum* and *P. vivax* prevalence rates by microscopy in households in close proximity to the Gilgel-Gibe dam [10]. However, the analysis of data from a 2-year (2008–2010) cohort study, involving children living around the same dam, and using an approach that does not account for recurrent episodes, was not able to show association between *P. falciparum* clinical malaria incidence and household distance to the dam [11].

Researchers have often used time-to-first event models to analyse malaria disease data from longitudinal studies [11–14]. Those models do not take into account that individuals can experience multiple episodes during the study period and ignore the probability of occurrence of a new episode possibly being influenced by earlier episodes [15]. Time-to-recurrent models, such as the frailty model used in the present study, have been applied to recurrent event data in several studies to accommodate for the correlation between event times within a same subject [16–18]. Those models have been used to estimate the intervention effects using recurrent episodes in clinical trials [19] as well as to assess risk factors for recurrent health conditions [20, 21].

Using a 2-year longitudinal malaria cohort data of children living nearby the Gilgel-Gibe dam in south-west Ethiopia, this study aimed to assess the effect of age, season period, and household distance to the dam on the species-specific clinical malaria incidence (*P. falciparum* and *P. vivax*) and to generate evidence to strengthen the prevention and control of malaria in the area. The study was implemented as part of several other studies, intended to assess the impact of the Gilgel-Gibe hydroelectric dam in the health and other sectors (environment, agriculture, economy, etc.) following its opening in 2004 [22].

## Methods

### Study area

**Fig. 1:**
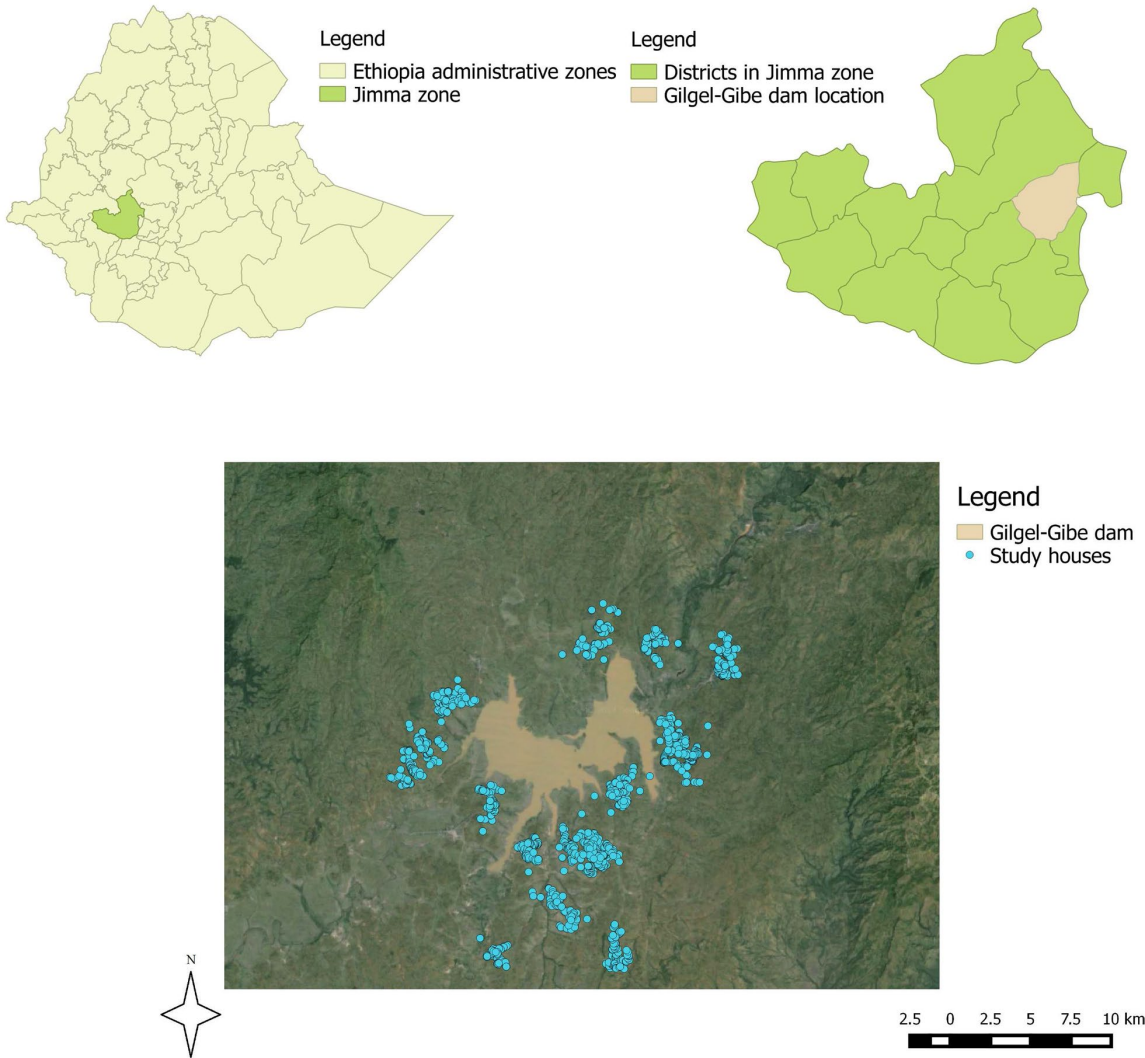
Map of the study area showing the Gilgel-Gibe hydroelectric dam reservoir, study villages and the distribution of study houses in south-western Ethiopia

The study area lies between latitudes 7°42′50″N and 07°53′50″N and between longitudes 37°11′22″E and 37°20′36″E at an altitude of 1734–1864 metres above sea level, and is located at 260 km south-west of the capital Addis Ababa in the Oromia region, south-western Ethiopia. The area is located near the Gilgel-Gibe hydroelectric dam, which covers a territory of 62 km^2^ (Fig. 1). The weather is sub-humid and warm, with an annual rainfall between 1300 and 1800 mm, and a mean annual temperature of 19 °C. Considering the rainfall pattern, three seasons can be identified along the year: the long rainy season from July to September, the dry season from October to March, and the moderate rainy season from April to June.

Sixteen villages within 10 km radius (265–9046 metres) from the dam reservoir shore were randomly selected based on similar eco-topography, access to health facilities, without major impounding water nearby and homogeneous with respect to socio-cultural and economic activities [11]. The main socio-economic local activities are mixed farming involving the cultivation of staple crops (maize, teff and sorghum), and cattle and small stock raising. All households in selected communities had access to health facilities and were socio-economically similar [10]. *Anopheles arabiensis* is the major local malaria vector [11].

### Study design and population

A longitudinal 2-year malaria cohort study was conducted in children under 10 years old, living in the selected 16 villages around the Gilgel-Gibe hydroelectric power dam. A total of 2040 children aged <10 years were enrolled in July 2008, and then weekly followed up until June 2010. Each child was identified with a unique code, and selected villages and households were geo-referenced using a handheld global positioning system (GPS) device (Garmin’s GPSMAP 60CSx, Garmin International Inc., USA), allowing the estimation of the household distance to the dam.

### Ethical approval

Ethical approval for the study was obtained from Jimma University Research and Ethics Committee. Verbal and written signed informed consent was obtained from the mother or caregiver of each child before enrollment of the child in the study.

### Malaria follow-up and laboratory processing

Active case detection (ACD) through weekly household visits allowed the identification and registration of all clinical malaria episodes in the study population during the follow-up period. During the household visits, axillary temperature was taken and the caregiver was asked about fever history. If a child had fever (temperature ≥37.5 °C) or reported a history of fever in the past 24 h, a finger-prick blood sample was taken for immediate diagnosis by microscopy in the same site or at Omo-Nada district Health Center Laboratory. Microscopy diagnosis was conducted by trained laboratory technicians. Thick smears were used to confirm the presence or absence of parasites, whereas the thin smear was used to identify the *Plasmodium* species.

All children with microscopically confirmed malaria were treated according to the national treatment guidelines [21]. Treatment was administered by the parents and/or guardians of the children, and consisted of 25 mg/kg of chloroquine (CQ) over three consecutive days for *P.vivax*, and artemether-lumefantrine (AL) for *P. falciparum* twice daily according to the body weight as follows: 5–14 kg, one tablet per dose; 15–24 kg, two tablets per dose; 25–34 kg, three tablets per dose; and adult, four tablets per dose. Treatment adherence was monitored during household visits by asking for the medication packages and the remaining pills.

Absent children were followed in the next visits and their caregivers were asked about the occurrence of symptomatic episodes and/or the confirmation of malaria by a health facility. In addition, all the health facilities near the study communities were monthly visited to verify their clinical records, checking if any enrolled children had presented a confirmed malaria episode in the past month not being detected during the weekly visits.

The monthly number of episodes by species and data published previously on the density of *A. arabiensis* during the study period were shown graphically. Mosquitoes were collected in all 16 communities, once a month, from 18:00 to 6:00 h in two selected houses using light trap catches. Further details on the mosquito collection methodology are described elsewhere [11].

### Data structure for modelling first and recurrent malaria episodes

Malaria episode data of each child were checked carefully to identify a first malaria episode as well as any additional malaria episodes after the first malaria episode (i.e. recurrent episodes). For the time at risk, a child treated for a malaria episode was censured for 21 days in order to prevent double counting of episodes and to allow for any prophylactic effect of the antimalarial treatment [24].

A sub-sample data of four children in Table 1 illustrates the differences in the data structures required for modelling first malaria episodes and recurrent malaria episodes. The time from the start of the follow-up (July 8th 2008) to the first episode (time-to-first episode) in children who presented malaria episodes, and the time to the last censorship in children who did not present malaria episodes was considered to model first malaria episodes. Therefore, this data structure excluded all additional information after the time of the first malaria episode. According to these criteria, from the sample of four children data layout (Table 1), only data from the first row of each child were used to model time-to-first episodes.

**Table 1:** Data structure showing a sample of four children malaria episode history (July 2008–June 2010) for the analysis of time-to-recurrent malaria episodes

ID	Start date	End date	Gap	Event	Sex	Age	Distance	Season
1	08/07/2008 *0*	04/11/2008 *119*	119	1	0	9	3.288	Dry
1	25/11/2008 *140*	10/06/2009 *337*	197	1	0	9	3.288	Long
1	01/07/2009 *358*	20/12/2009 *530*	172	1	0	9	3.288	Dry
1	10/01/2010 *551*	04/06/2010 *696*	145	0	0	9	3.288	Long
2	08/07/2008 *0*	07/10/2008 *91*	91	1	1	3	8.776	Dry
2	28/10/2008 *112*	14/11/2008 *129*	17	1	1	3	8.776	Dry
2	05/12/2008 *150*	04/06/2010 *696*	543	0	1	3	8.776	Long
3	08/07/2008 *0*	19/09/2008 *73*	73	1	1	3	7.029	Long
3	10/10/2008 *94*	29/05/2010 *690*	596	0	1	3	7.029	Moderate
4	08/07/2008 *0*	04/06/2010 *696*	696	0	0	5	5.287	Long

**Fig. 2:**
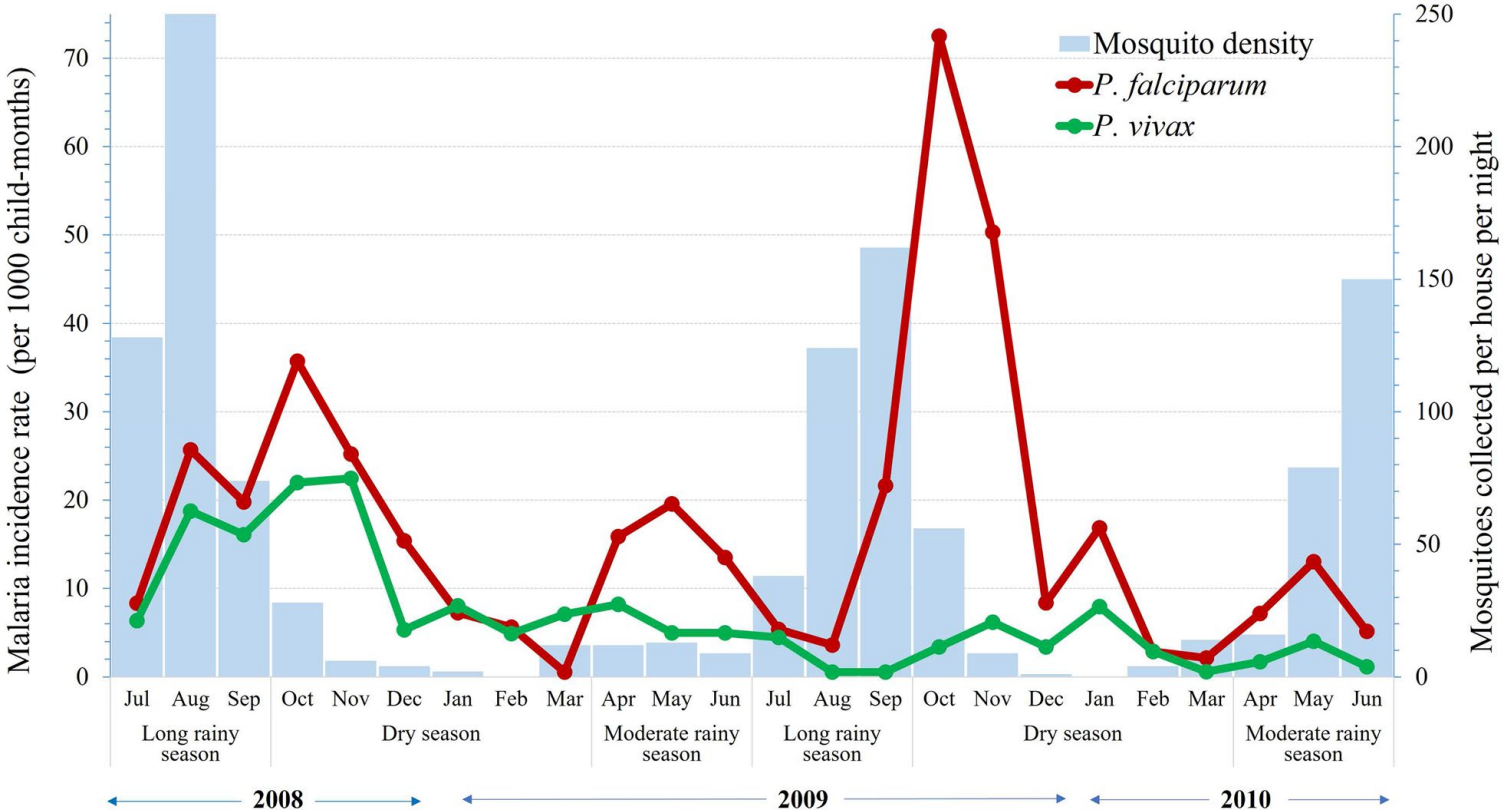
Monthly malaria episodes in study children in south-western Ethiopia (July 2008–June 2010)

Conversely, data from children with both one and more malaria episodes were accounted when modelling recurrent episodes. The time at risk for a recurrent episode in each child with recurrent episodes was given by the time between two successive malaria episodes minus 21 days (i.e. a discontinuous time interval data structure). For instance, the follow-up history of one child is described by the first four rows of Table 1. The first row indicates that the child began the follow-up at time 0 and remained at risk of presenting a malaria episode until day 119 when an episode was confirmed; the second row indicates that the risk of presenting another episode in the child re-started 21 days after a previous episode at day 140 and ended on day 337 when the episode occurred; the third row indicates that the risk re-started at day 358 and ended on day 530; and the fourth row indicates that the child was followed until day 696, no longer presenting additional malaria episodes. The second child had two malaria episodes at day 91 and 129 and the follow-up ended on day 696, the third child had one malaria episode at day 73 and the follow-up ended on day 690 without a malaria episode, and the fourth child had no episodes during the follow-up period. Additional file 1 shows the follow-up history of a sample of four children and the data structure representation for time-to-recurrent episode models according to a counting process (calendar timescale). Figure 2a shows the children follow-up time line; one time line represents the total follow-up period history. In the calendar time layout (Fig. 2b), each time line represents the length of time at risk for a new malaria episode in the same child.

### Statistical analysis

Malaria incidence rates (by species) were calculated by dividing the number of episodes during the study period by child-months at risk, and expressed as the number of episodes per 1000 child-months at risk. A child-month at risk corresponds to a child that had at least once weekly visit of the total 4 weeks of a specific month. Correspondingly, 95 % confidence intervals (95 % CI) of the incidence rates were calculated assuming that the number of new malaria episodes was Poisson distributed.

Frailty models were applied to calculate hazard ratios (HRs) of species-specific incidence using separately time-to first episode data and time-to-recurrent episode data. The following potential risk factors were included in the univariate and multivariate models: gender, age groups (≤3, 3–7, ≥7 years), household distance to the dam reservoir (in kilometres), and season (long rainy season, dry season, and moderate rainy season). For covariates with more than two categories, a global single *p* value was calculated, using a Wald test. Models were fitted in R version 3.2 using the package ‘frailtypack 2.5.1’ [25].

The time-to-first episode data was modelled through a piecewise Weibull frailty model. The piecewise interval was introduced taking into account the hazard rate changes in malaria incidence between the different seasons in the 2 years (thus in total six constants, one for each season and year combination). This flexible parametric approach has been used in a previous study aiming to model malaria recurrent events, with good results [24]. The two parameter model is given by the formula $h\left( t \right) = \lambda \rho t^{\wedge } \left( {\rho - 1} \right)$; where *λ* (lambda) is the scale parameter, and *ρ* (rho) the shape parameter.

The time-to-recurrent malaria episode data was modelled by nested frailty models allowing to account for the hierarchical clustering of the data by including two nested random effects that act multiplicatively on the hazard function [25], using a calendar timescale with discontinuous time interval data structure. In this study, village was introduced as a cluster while children within each village were considered as a sub-cluster. The cluster “village” random effect variance was estimated by *Ɵ* (theta) while the sub-cluster “individual” random effect variance was estimated by *η* (eta) from the nested frailty model. For model checking, martingale residual of the frailty model were plotted against covariates at a continuous level, confirming the fit of the data when no systematic or clear pattern is observed [26].

## Results

The 2040 children, followed-up from 2008 to June 2010, accounted for 46,972 child-months at risk. Male children (51.9 %) slightly outnumbered female children (48.1 %), and the median age at the time of enrolment was 5 years. A total of 1070 clinical malaria episodes were registered during the study period, including 685 episodes of *P. falciparum* in 548 children (421 with one episode, 119 with two, seven with three, and one with five episodes) and 385 episodes of *P. vivax* in 316 children (263 with one episode, 40 with two, 10 with three, and three with four episodes). *Plasmodium falciparum* and *P. vivax* malaria incidence rates were 14.6 (95 % CI: 13.4–15.6) and 8.2 (95 % CI: 7.3–9.1) per 1000 children per month, respectively. *Plasmodium falciparum* episodes predominated over *P. vivax* episodes in all selected communities (Additional file 2); episodes by both species peaked during the dry season after an increase of the mosquito density in the previous months (Fig. 2). While *P. vivax* episodes were more frequent in children younger than 3 years of age (p < 0.001), *P. falciparum* episodes occurred mostly in children older than 7 years (p < 0.001) (Table 2). Differences between boys and girls in the number of species-specific malaria episodes were not statistically significant (p > 0.05).

**Table 2:** Characteristics of the study children by number of malaria episodes

Characteristics	Malaria episodes, N (%)				
	1	2	3	4	None (censored)
For *P. vivax* episodes					
Gender					
Male (N = 1059)	129 (12.2)	25 (2.3)	4 (0.4)	2 (0.2)	899 (84.9)
Female (N = 981)	130 (13.3)	10 (1.0)	6 (0.6)	1 (0.1)	834 (85.0)
Age					
≤3 years (N = 974)	155 (15.9)	15 (1.6)	6 (0.6)	1 (0.1)	797 (81.8)
3–7 years (N = 856)	86 (10.1)	18 (2.1)	2 (0.2)	2 (0.2)	748 (87.4)
≥7 years (N = 210)	18 (8.5)	2 (1.0)	2 (1.0)	0 (0.0)	188 (89.5)
For *P. falciparum* episodes					
Gender					
Male (N = 1059)	212 (20.0)	51 (4.8)	5 (0.5)	1 (0.1)^a^	790 (74.6)
Female (N = 981)	209 (21.3)	68 (5.9)	2 (0.2)	–	702 (71.6)
Age					
≤3 years (N = 974)	213 (21.9)	57 (5.9)	1 (0.1)	0 (0.0)	703 (72.1)
3–7 years (N = 856)	170 (19.9)	45 (5.3)	2 (7.1)	0 (0.0)	639 (74.7)
≥7 years (N = 210)	38 (18.1)	17 (8.1)	4 (1.9)	1 (0.5)	150 (71.4)

^a^This child had an additional episode (5th episode)

Table 3 shows the follow-up-time and numbers of children included in the time-to-first episode models and in the time-to-recurrent episode models. The median time to the first episode in 316 children (134 days) who had at least one *P. vivax* episode throughout the study period was slightly lower than the median time between the first and second episodes in 53 children (147 days), and between the second and third episodes in 13 children (172 days). On the other hand, the median time to the first episode in 548 children who had at least one *P. falciparum* episode (286 days) were higher than the median time between the first and second episodes in 127 children (204 days), and between the second and third episodes in eight children (131 days).

**Table 3:** Number of children by the number of episodes for both species, along with time included in time-to-first event and time-to-recurrent event models

	Follow-up			
	Until 1st episode	Between 1st and 2nd episodes	Between 2nd and 3rd episodes	Between 3rd and 4th episodes
For *P. vivax* episodes				
Number of followed-up children				
Total (N)	2040	316	53	13
With episodes	316	53	13	3
Censored	1724	263	40	10
Follow-up time				
Mean (days)	221	208	196	139
Median (days)	134	147	172	99
[Min.–max.]	[7–696]	[9–621]	[49–402]	[66–180]
For *P. falciparum* episodes				
Number of followed-up children				
Total (N)	2040	548	127	8
With episodes	548	127	8	1^a^
Censored	1492	421	119	7
Follow-up time				
Mean (days)	283	233	133	111
Median (days)	286	204	131	105
[Min.–max.]	[7–696]	[9–639]	[66–339]	[63–189]

^a^This child had an additional episode (5th episode)

Multivariate time-to-first episode and time-to-recurrent episode models showed (using global and local p-values) that the age of the child at enrolment and season were significantly associated with the incidence of *P. vivax* episodes even after controlling for other non-significant potential risk factors such as gender and household proximity to the dam (Tables 4 and 5). While adjusted hazard ratios (AHR), adjusted for age groups were similar in both models indicating that children aged >3 years old were at lower risk of presenting *P. vivax* episodes than younger children (AHR ~ 0.7; 95 % CI: ~0.5–0.9), AHR estimates for the effect of season categories differed slightly between models. *P. vivax* episodes occurred 1.9 times more frequently in the dry season than in the long rainy season according to the time-to-first event model (AHR = 1.9; 95 % CI: 1.2–2.9) (Table 4), while such episodes were estimated to be 2.7 times higher in the dry season than in the long rainy season (AHR = 2.7; 95 % CI: 2.2–3.5) (Table 5), when using time-to-recurrent event model.

**Table 4:** Univariate and multivariate adjusted risk factors analysis for time-to-first *P. vivax* episode and *P. falciparum* episode

	*P. vivax*			*P. falciparum*		
	Univariate HR (95 % CI)	Multivariate AHR (95 % CI)	G p-value	Univariate HR (95 % CI)	Multivariate AHR (95 % CI)	G p-value
Gender						
Female	1	1		1	1	
Male	1.0 (0.8,1.2)	0.9 (0.8,1.2)		0.9 (0.8,1.1)	0.9 (0.8,1.1)	
Age (years)						
≤3	1	1	0.01*	1	1	<0.01*
3–7	0.8* (0.6,1.0)	0.7* (0.6,0.9)		1.2 (1.0,1.5)	1.2* (1.0,1.5)	
≥7	0.6 (0.4,1.0)	0.6* (0.4,0.9)		1.4* (1.1,1.9)	1.4* (1.2,2.2)	
Proximity to the dam						
≤1.17 km	1	1	0.12	1	1	0.53
1.17–1.79	0.8 (0.6,1.2)	0.9 (0.6,1.2)		0.9 (0.7,1.2)	1.0 (0.7,1.2)	
1.79–2.97	0.9 (0.6,1.3)	0.8 (0.5,1.3)		0.9 (0.7,1.2)	1.0 (0.7,1.3)	
>2.97	0.8 (0.5,1.5)	0.8 (0.5,1.4)		1.1 (0.8,1.7)	1.1 (0.8,1.5)	
Season						
Long rainy	1	1	<0.01*	1	1	<0.01*
Dry	1.7* (1.1,2.3)	1.9* (1.2,2.9)		1.7* (1.2,2.8)	3.2 (2.2,5.2)	
Moderate rainy	0.8 (0.7,1.1)	0.8 (0.6,1.2)		1.2* (1.0,2.0)	2.3* (1.4,3.1)	

*HR* hazard ratio; *AHR* adjusted hazard ratio; *G p-value* global p-value

* P < 0.05

**Table 5:** Univariate and multivariate adjusted risk factors analysis for time-to-recurrent *P. vivax* episodes and *P. falciparum* episodes

	*P. vivax*			*P. falciparum*		
	Univariate HR (95 % CI)	Multivariate AHR (95 % CI)	G P-value	Univariate HR (95 % CI)	Multivariate AHR (95 % CI)	G P-value
Gender						
Female	1	1		1	1	
Male	1.0 (0.8,1.2)	1.0 (0.8,1.2)		0.9 (0.8,1.1)	0.9 (0.7,1.0)	
Age (years)						
≤3	1	1	<0.01*	1	1	0.02*
3–7	0.8 (0.6,1.0)	0.7* (0.6,0.9)		1.2 (0.9,1.4)	0.9 (0.8,1.1)	
≥7	0.6* (0.4,0.9)	0.6* (0.4,0.9)		1.7* (1.3,2.2)	1.2* (1.1,1.6)	
Proximity to the dam						
≤1.17 km	1	1	0.25	1	1	0.91
1.17–1.79	0.8 (0.7,1.1)	0.8 (0.6,1.1)		0.9 (0.8,1.1)	0.9 (0.7,1.2)	
1.79–2.97	0.9 (0.7,1.1)	0.9 (0.7,1.2)		1.0 (0.8,1.1)	1.0 (0.8,1.3)	
>2.97	1.0 (0.8,1.3)	0.9 (0.7,1.2)		1.1 (0.9,1.3)	1.1 (0.9,1.3)	
Season						
Long rainy	1	1	<0.01*	1	1	<0.01*
Dry	2.9* (2.2,3.9)	2.7* (2.2,3.5)		14.6* (12.0,17.7)	16.9 (14.3,20.2)	
Moderate rainy	0.9 (0.6,1.2)	1.0 (0.8,1.3)		1.1 (0.8,1.4)	1.4* (1.1,1.8)	
Model parameters						
*ρ*		0.75			0.86	
*Ɵ* (SE)		0.36 (0.13)			0.58 (0.27)	
*η* (SE)		0.62 (0.26)			0.46 (0.19)	

*HR* hazard ratio; *AHR* adjusted hazard ratio; *SE* standard error, *G p-value* global p-value

* P < 0.05

Similarly to *P. vivax* models, both types of multivariate models for *P. falciparum* also found that age and season were significantly associated with the incidence of *P. falciparum* episodes, but with differences in AHR estimates (Tables 4 and 5). For the time-to-first episode model, children aged 3–7 years (AHR = 1.2; 95 % CI: 1.0–1.5) and those older than 7 years (AHR = 1.4; 95 % CI: 1.2–2.2) were at greater risk of having *P. falciparum* episodes than children aged ≤3 years (Table 4). Conversely, for the time-to-recurrent episode model, children older than 7 years had higher hazards of new *P. falciparum* episodes in comparison to children younger than or equal to 3 years (AHR = 1.2; 95 % CI: 1.1–1.6) (Table 5). Although *P.falciparum* episodes were most common in dry and moderate seasons according to both models, AHR estimates resulting from time-to-recurrent episode model for the dry season compared to the long rainy season were considerably higher (AHR = 16.9; 95 % CI: 14.3–20.2) than the ones obtained from the time-to-first episode model (AHR = 2.7; 95 % CI: 2.2–3.5). Gender and distance to the dam were not significantly associated with *P. falciparum* incidence in both models. Regarding model parameter estimates, *ρ* values lower than one, indicated that for both species incidence rates decreased with time, but incidence rates for *P. vivax* (*ρ* ~ 0.7) during the study period decreased faster than those for *P. falciparum* (*ρ* ~ 0.8). The malaria risk variation among villages for *P. falciparum* (*ɵ* ~ 0.5) was higher than for *P. vivax* (*ɵ* ~ 0.3). Moreover, malaria risk variation among children (*η*) were higher for *P. vivax* than for *P. falciparum*. Additional file 3 shows the fit of the data by plotting martingale residual by the covariate at a continuous level. The lowess estimates did not show a clear pattern, suggesting a good fit.

## Discussion

This study reports species-specific clinical malaria episode patterns in a 2-year longitudinal cohort study of 2040 children under 10 years old living nearby the Gilgel-Gibe hydropower dam, in south-western Ethiopia. Multiple *P. falciparum* and *P. vivax* malaria episodes occurred in respectively 127 and 53 children along the study period. *Plasmodium vivax* clinical episodes were more obvious in younger age groups and occurred mostly in the dry season, while *P. falciparum* episodes predominated in older children and occurred in the dry and moderate season but with a substantial peak in the first months of the dry season. Both *P. vivax* and *P. falciparum* incidence rates decreased with time and were not associated with the household proximity to the dam reservoir.

This is the first study analysing recurrent malaria episodes with two statistical approaches, i.e. time-to-first event models and time-to-recurrent event models with discontinuous risk intervals. Although no major differences in parameter estimates for evaluating risk factors were found between both models using the data at hand, it is not the rule for all health conditions with recurrent episodes and possibly mainly explained by the low occurrence of recurrent malaria episodes in the study. Indeed, a re-analysis of data from a pneumococcal conjugate vaccine trial and the simulation of scenarios found that the difference between effect estimates of the vaccine obtained from time-to-first event models and time-to-recurrent event models was substantial especially when a high incidence of recurrent episodes was noted [27]. Moreover, a number of studies dealing with recurrent health conditions have observed different results when applying both models separately [15, 24, 26, 28].

The shape model parameter value below one confirmed that malaria incidence rates for both species decreased with time during the study period. A similar decreasing malaria incidence trend has been reported in southwest Ethiopia [29] and may be explained by positive effects of control interventions in the area. Indeed, the government re-started indoor residual spraying with deltamethrin in 2009 and replaced old long-lasting insecticidal nets (LLINs) in 2010 [30].

Although previous studies in Ethiopia and other African countries have reported dams influencing malaria transmission by increasing vector abundance due to breeding sites associated with dam´s floods on the shorelines [6, 31–33], the present study did not identify an association between the proximity to the Gilgel-Gibe dam reservoir and the species-specific clinical malaria incidence. The design and the automatic operation of this dam may prevent the appearance of shoreline puddles and consequently the formation of breeding sites near the dam [32]. On the other hand, agriculture field puddles, wet lands, man-made pools, and rain pools located extensively in all the study area would become the most important sources of mosquitoes, and these sources may significantly enlarge after the long rainy season. In addition, since the main economic activities in the communities are related to cattle, animal hoof-prints filled with water would emerge after rains, thus facilitating the development of mosquitoes [34].

The differences in clinical malaria incidence between species with respect to age may be related to a different species-specific acquisition rate of immunity in the Ethiopian children, which may be related to different exposure levels for both species in the early years of live. Those differences are in line with previous findings from longitudinal studies conducted in co-endemic malaria areas of Papua New Guinea [8, 14, 35]. Malarial immunity in New Guinean children is acquired much more rapidly with *P. vivax* than with *P. falciparum*. After 5 years of continuous exposure, children can acquire an almost complete clinical immunity to *P. vivax*, which is characterized by their control of blood-stage parasite densities rather than the acquisition of a significant immunity against infection [14, 35]. This differential pattern between species was also reported in low transmission setting. In longitudinal studies conducted in Thailand [36, 37], and in Vanuatu [38] the incidence of *P. vivax* malaria also decreased significantly faster with age than that the incidence of *P. falciparum*, while a cohort study in the Brazilian Amazon indicated that *P. vivax* malaria started to decrease after 5–6 years of residence in the endemic area compared to 8–9 years for *P. falciparum* [39].

Although a weekly identification of symptomatic children and the consequent diagnosis and treatment of malaria infections during the study are likely to have contributed to the decrease of malaria transmission in the area and consequently possibly explain the decrease of the malaria incidence rate in time, it is worth to notice that the decrease may also be associated with the acquisition of immunity in the study children. Further longitudinal studies incorporating the detection and follow-up of both symptomatic and asymptomatic infections in young children are necessary to understand the role of immunity (i.e. immunity to infection and/or clinical immunity) in the malaria transmission in the co-endemic Ethiopia [8, 14, 35].

Many studies have reported high mosquito densities and an increased malaria risk following the rainy season [5, 40]. After rainy periods, intermittent streams could create pockets or pools of water which can serve as potential breeding sites for mosquitoes, contributing to an increase in mosquito density and vector-human contacts; therefore contributing to a greater number of malaria episodes during the dry season. Although malaria is transmitted by both species in the study, it occurred seasonally and mainly after increased rainfall, *P. vivax* clinical episodes being less sensitive to seasonal and environmental changes than *P. falciparum*, an important proportion of the former possibly being hypnozoite-triggered relapses [7–9]. This hypothesis is further supported by the fact that children with confirmed *P. vivax* episodes only received CQ, following the national guidelines for areas where the glucose-6-phosphate dehydrogenase deficiency (G6PD deficiency) is not known and where tests to detect that condition are not available [23]. A previous study that used a life-table method showed that in Ethiopia the cumulative risk of recurrent episodes at day 157 was significantly higher in the CQ group (61.8 %), compared with the CQ + PQ (primaquine) group (26.3 %) [41].

## Conclusion

The analysis of all malaria episodes (first and recurrent episodes) found different species-specific patterns of malaria disease in the enrolled children, with mild seasonality in the incidence of *P. vivax* episodes, mostly seen in younger age groups, and with marked seasonality in the incidence of *P. falciparum* episodes mainly observed in older children. The decision-making and planning of malaria control interventions will have to consider the seasonal patterns of malaria transmission in order to reduce malaria incidence, as well as a species-specific policy.

## Additional files

10.1186/s12936-016-1253-2 Recurrent malaria episode data representation for a sample of four children: (a) four children follow-up history; (b) children malaria episode information using calendar timescale or counting process approach.
10.1186/s12936-016-1253-2 *Plasmodium falciparum* and *Plasmodium vivax* episodes by village in south western Ethiopia (July 2008–June 2010).
10.1186/s12936-016-1253-2 Martingal residual plots with corresponding lowess estimates (red line) (a) for *P. vivax*, (b) for *P. falciparum.*

## References

[1] WHO (2015). World Malaria Report 2015.

[2] Nigatu W, Abebe M, Dejene A (1992). *Plasmodium vivax* and *P. falciparum* epidemiology in Gambella, south-west Ethiopia. Trop Med Parasitol.

[3] Gething PW, Elyazar IRF, Moyes CL, Smith DL, Battle KE, Guerra CA (2012). A long neglected world malaria map: *Plasmodium vivax* endemicity in 2010. PLoS Negl Trop Dis.

[4] Olana D, Chibsa S, Teshome D, Mekasha A, Graves PM, Reithinger R (2011). Malaria, Oromia regional state, Ethiopia, 2001–2006. Emerg Infect Dis.

[5] Woyessa A, Deressa W, Ali A, Lindtjørn B (2012). Prevalence of malaria infection in Butajira area, south-central Ethiopia. Malar J.

[6] Ghebreyesus TA, Haile M, Witten KH, Getachew A, Yohannes M, Lindsay SW (2000). Household risk factors for malaria among children in the Ethiopian highlands. Trans R Soc Trop Med Hyg.

[7] White NJ (2011). Determinants of relapse periodicity in *Plasmodium vivax* malaria. Malar J.

[8] Mueller I, Galinski MR, Baird JK, Carlton JM, Kochar DK, Alonso PL (2009). Key gaps in the knowledge of *Plasmodium vivax*, a neglected human malaria parasite. Lancet Infect Dis.

[9] Wells TNC, Burrows JN, Baird JK (2010). Targeting the hypnozoite reservoir of *Plasmodium vivax*: the hidden obstacle to malaria elimination. Trends Parasitol.

[10] Yewhalaw D, Legesse W, Van Bortel W, Gebre-Selassie S, Kloos H, Duchateau L (2009). Malaria and water resource development: the case of Gilgel-Gibe hydroelectric dam in Ethiopia. Malar J.

[11] Yewhalaw D, Getachew Y, Tushune K, Michael WK, Kassahun W, Duchateau L (2013). The effect of dams and seasons on malaria incidence and anopheles abundance in Ethiopia. BMC Infect Dis.

[12] Stefani A, Hanf M, Nacher M, Girod R, Carme B (2011). Environmental, entomological, socioeconomic and behavioural risk factors for malaria attacks in Amerindian children of Camopi, French Guiana. Malar J.

[13] Le Port A, Cottrell G, Martin-Prevel Y, Migot-Nabias F, Cot M, Garcia A (2012). First malaria infections in a cohort of infants in Benin: biological, environmental and genetic determinants. Description of the study site, population methods and preliminary results. BMJ Open.

[14] Michon P, Cole-Tobian JL, Dabod E, Schoepflin S, Igu J, Susapu M (2007). The risk of malarial infections and disease in Papua New Guinean children. Am J Trop Med Hyg.

[15] Ullah S, Gabbett TJ, Finch CF (2014). Statistical modelling for recurrent events: an application to sports injuries. Br J Sports Med.

[16] Rondeau V, Mazroui Y, Gonzalez J (2012). Frailtypack: an R package for the analysis of correlated survival data with frailty models using penalized likelihood estimation. J Stat Softw.

[17] Liu L, Wolfe RA, Huang X (2004). Shared frailty models for recurrent events and a terminal event. Biometrics.

[18] Duchateau L, Janssen P (2007). The Frailty model.

[19] Parpia S, Thabane L, Julian JA, Whelan TJ, Levine MN (2013). Empirical comparison of methods for analyzing multiple time-to-event outcomes in a non-inferiority trial: a breast cancer study. BMC Med Res Methodol.

[20] Duchateau L, Janssen P, Kezic I, Fortpied C (2003). Evolution of recurrent asthma event rate over time in frailty models. Appl Statist.

[21] Gabbett TJ, Ullah S, Finch CF (2012). Identifying risk factors for contact injury in professional rugby league players–application of a frailty model for recurrent injury. J Sci Med Sport.

[22] http://www.vliruos.be/media/74091/mid_term_evaluation_jimma_university.pdf.

[23] FDRE (2012). Ministry of Health: National Malaria Guideline.

[24] Sagara I, Giorgi R, Doumbo OK, Piarroux R, Gaudart J (2014). Modelling recurrent events: comparison of statistical models with continuous and discontinuous risk intervals on recurrent malaria episodes data. Malar J.

[25] Rondeau V, Filleul L, Joly P (2006). Nested frailty models using maximum penalized likelihood estimation. Stat Med.

[26] Twisk JW, Smidt N, de Vente W (2005). Applied analysis of recurrent events: a practical overview. J Epidemiol Community Health.

[27] Cheung YB, Xu Y, Tan SH, Cutts F, Milligan P (2010). Estimation of intervention effects using first or multiple episodes in clinical trials: the Andersen-Gill model re-examined. Stat Med.

[28] Guo Z, Gill TM, Allore HG (2008). Modeling repeated time-to-event health conditions with discontinuous risk intervals: an example of a longitudinal study of functional disability among older persons. Methods Inf Med.

[29] Sena L, Deressa W, Ali A (2014). Dynamics of *Plasmodium falciparum* and *Plasmodium vivax* in a micro-ecological setting, Southwest Ethiopia: effects of altitude and proximity to a dam. BMC Infect Dis.

[30] MOH (2012). National malaria guidelines.

[31] Lautze J, McCartney M, Kirshen P, Olana D, Jayasinghe G, Spielman A (2007). Effect of a large dam on malaria risk: the Koka reservoir in Ethiopia. Trop Med Int Health.

[32] Kibret S, McCartney M, Lautze J, Jayasinghe G (2009). Malaria transmission in the vicinity of impounded water: evidence from the Koka Reservoir. Ethiopia. IWMI Res Rep.

[33] Kibret S, Lautze J, McCartney M, Wilson GG, Nhamo L (2015). Malaria impact of large dams in sub-Saharan Africa: maps, estimates and predictions. Malar J.

[34] Mayagaya VS, Nkwengulila G, Lyimo IN, Kihonda J, Mtambala H, Ngonyani H (2015). The impact of livestock on the abundance, resting behaviour and sporozoite rate of malaria vectors in southern Tanzania. Malar J.

[35] Lin E, Kiniboro B, Gray L, Dobbie S, Robinson L, Laumaea A (2010). Differential patterns of infection and disease with *P. falciparum* and *P. vivax* in young Papua New Guinean children. PLoS One.

[36] Lawpoolsri S, Chavez IF, Yimsamran S, Puangsa-Art S, Thanyavanich N, Maneeboonyang W (2010). The impact of human reservoir of malaria at a community-level on individual malaria occurrence in a low malaria transmission setting along the Thai-Myanmar border. Malar J.

[37] Phimpraphi W, Paul RE, Yimsamran S, Puangsa-art S, Thanyavanich N, Maneeboonyang W (2008). Longitudinal study of *Plasmodium falciparum* and *Plasmodium vivax* in a Karen population in Thailand. Malar J.

[38] Maitland K, Williams TN, Bennett S, Newbold CI, Peto TE, Viji J (1996). The interaction between *Plasmodium falciparum* and *P. vivax* in children on Espiritu Santo island, Vanuatu. Trans R Soc Trop Med Hyg.

[39] da Silva-Nunes M, Codeço CT, Malafronte RS, da Silva NS, Juncansen C, Muniz PT (2008). Malaria on the Amazonian frontier: transmission dynamics, risk factors, spatial distribution, and prospects for control. Am J Trop Med Hyg.

[40] Abate A, Degarege A, Erko B (2013). Community knowledge, attitude and practice about malaria in a low endemic setting of Shewa Robit Town, northeastern Ethiopia. BMC Public Health.

[41] Yeshiwondim AK, Tekle AH, Dengela DO, Yohannes AM, Teklehaimanot A (2010). Therapeutic efficacy of chloroquine and chloroquine plus primaquine for the treatment of *Plasmodium vivax* in Ethiopia. Acta Trop.

