# Supplementary figures and images for: Identification of different malaria patterns due to Plasmodium falciparum and Plasmodium vivax in Ethiopian children: a prospective cohort study
Source: Malar J. 2016 Apr 14;15:208. doi: 10.1186/s12936-016-1253-2 (PMC4831103; doi:10.1186/s12936-016-1253-2)

Children

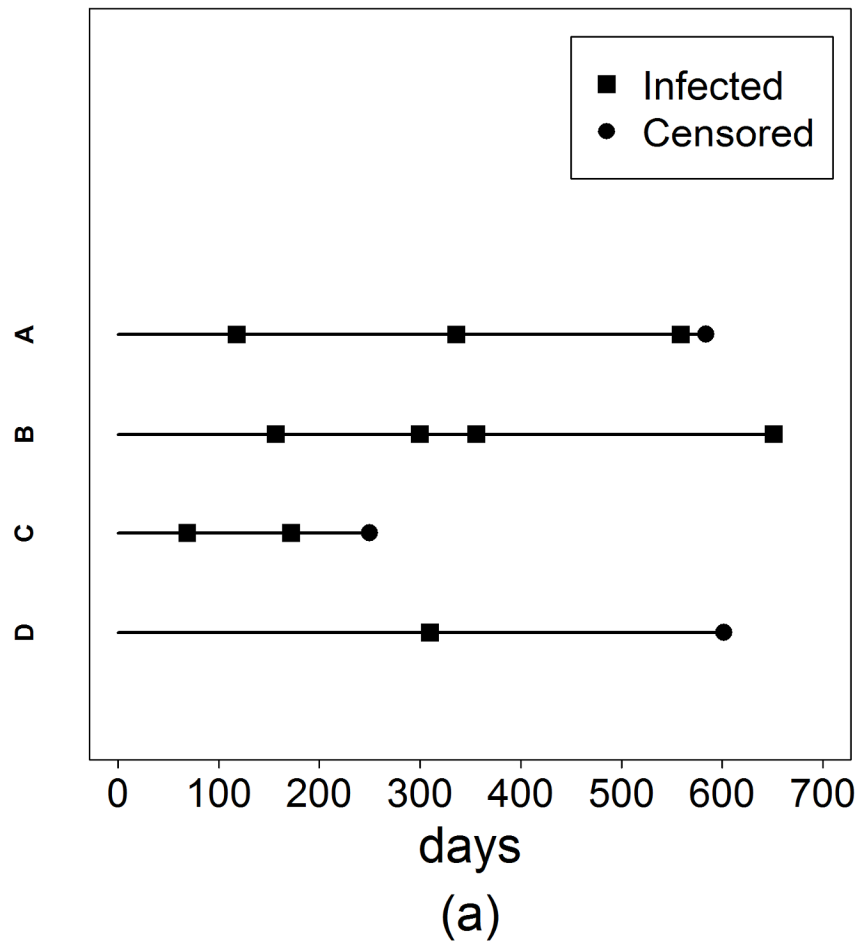

Children

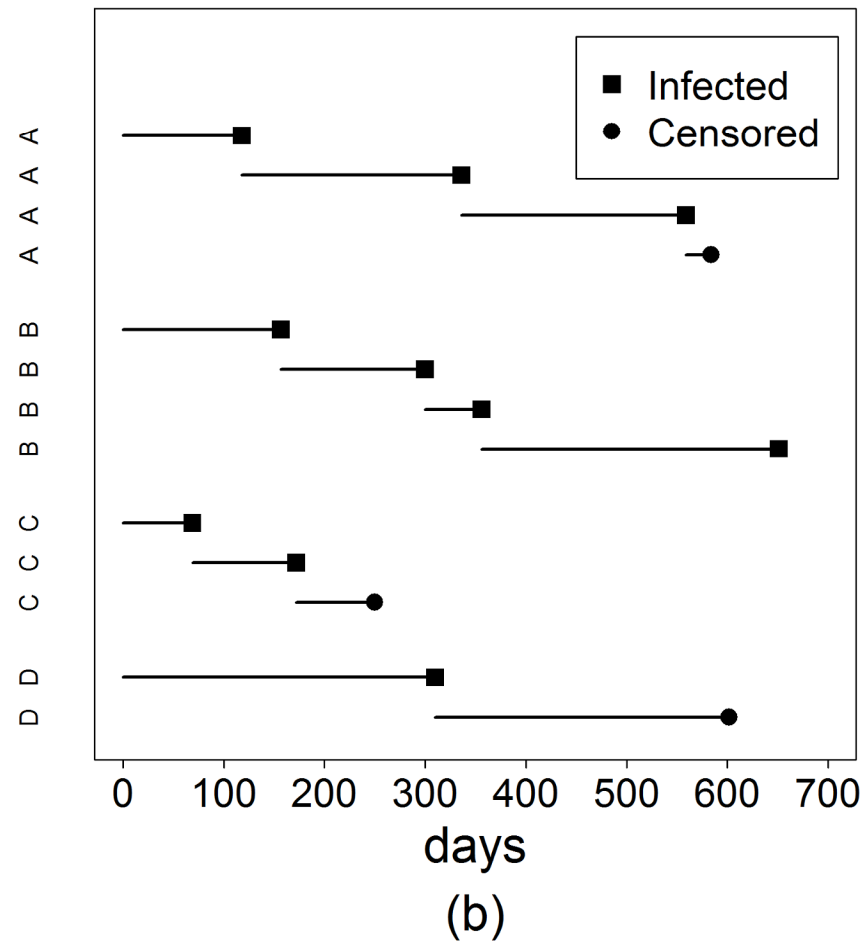

Supplement: Supplementary file 1 — 10.1186/s12936-016-1253-2 Recurrent malaria episode data representation for a sample of four children: (a) four children follow-up history; (b) children malaria episode information using calendar timescale or counting process approach. [file 12936_2016_1253_MOESM1_ESM.pdf]

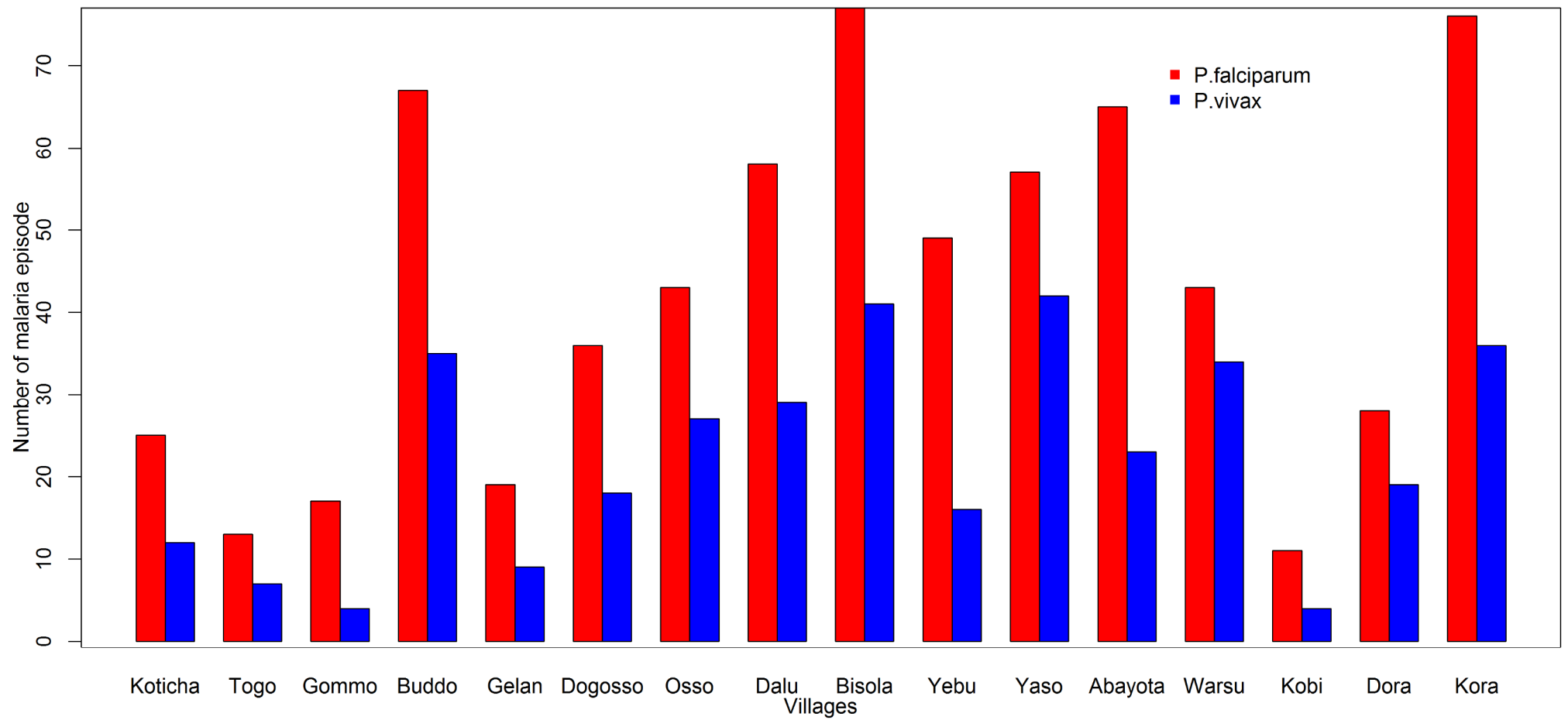

Supplement: Supplementary file 2 — 10.1186/s12936-016-1253-2 Plasmodium falciparum and Plasmodium vivax episodes by village in south western Ethiopia (July 2008–June 2010). [file 12936_2016_1253_MOESM2_ESM.pdf]

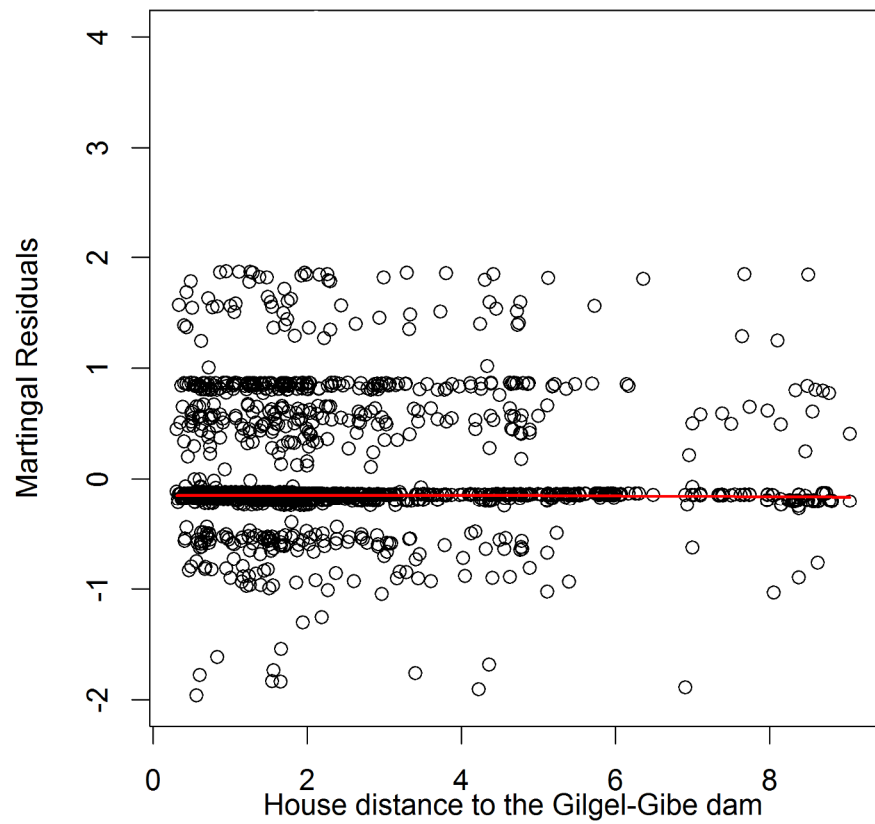

(a)

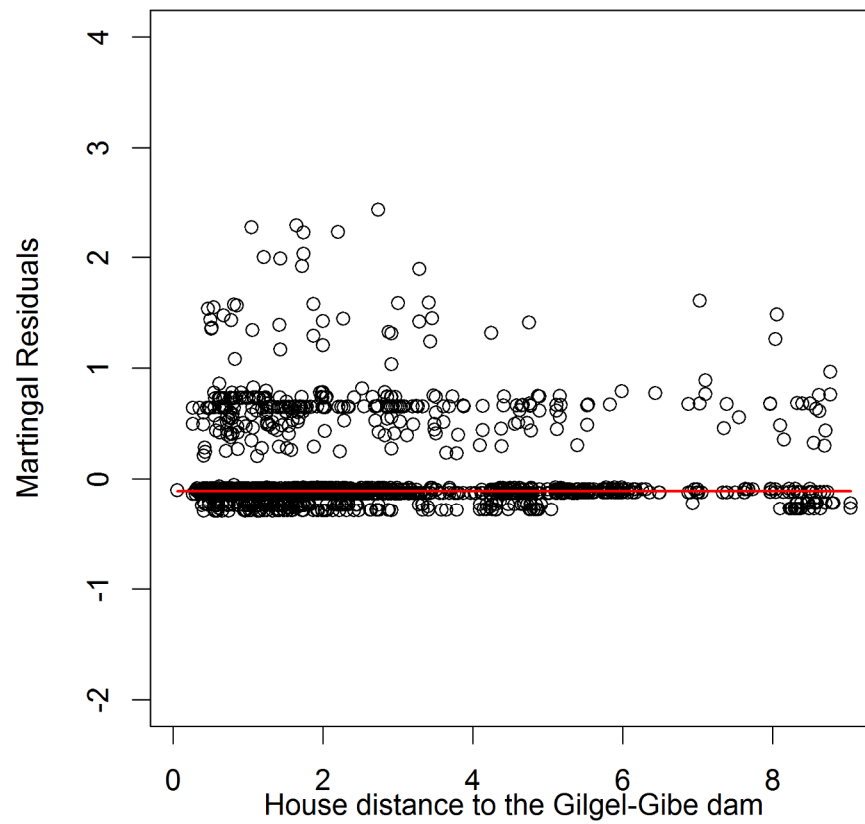

(b)

Supplement: Supplementary file 3 — 10.1186/s12936-016-1253-2 Martingal residual plots with corresponding lowess estimates (red line) (a) for P. vivax, (b) for P. falciparum. [file 12936_2016_1253_MOESM3_ESM.pdf]
